# Impact-induced initiation of Snowball Earth: A model study

**DOI:** 10.1126/sciadv.adk5489

**Published:** 2024-02-09

**Authors:** Minmin Fu, Dorian S. Abbot, Christian Koeberl, Alexey Fedorov

**Affiliations:** ^1^ Department of Earth and Planetary Sciences, Yale University, 210 Whitney Ave., New Haven, CT 06511, USA.; ^2^ Department of Geophysical Sciences, University of Chicago, 5734 S Ellis Ave., Chicago, IL 60637, USA.; ^3^ Department of Lithospheric Research, University of Vienna, Althanstrasse 14, 1090 Vienna, Austria.

## Abstract

During the Neoproterozoic and Paleoproterozoic eras, geological evidence points to several “Snowball Earth” episodes when most of Earth’s surface was covered in ice. These global-scale glaciations represent the most marked climate changes in Earth’s history. We show that the impact winter following an asteroid impact comparable in size to the Chicxulub impact could have led to a runaway ice-albedo feedback and global glaciation. Using a state-of-the-art atmosphere-ocean climate model, we simulate the climate response following an impact for preindustrial, Last Glacial Maximum (LGM), Cretaceous-like, and Neoproterozoic climates. While warm ocean temperatures in the preindustrial and Cretaceous-like climates prevent Snowball initiation, the colder oceans of the LGM and cold Neoproterozoic climate scenarios rapidly form sea ice and demonstrate high sensitivity to the initial condition of the ocean. Given suggestions of a cold pre-Snowball climate, we argue the initiation of Snowball Earth by a large impact is a robust possible mechanism, as previously suggested by others, and conclude by discussing geologic tests.

## Teaser

A large impact event during a cold climate, such as the last ice age, could have tipped Earth into a totally ice-covered state.

## INTRODUCTION

During the Cryogenian period of the Neoproterozoic (720 to 635 Ma), multiple lines of evidence point to at least two “Snowball Earth” episodes when ice extended to the equator (*1*, *2*). The onset of the first event, the Sturtian glaciation, has been dated to 717.5 to 716.3 Ma, while the second, the Marinoan glaciation, began around 650 to 639 Ma (*3*–*5*). A number of glacial deposits have also been identified during the Paloeoproterozoic (*6*), with at least one of them interpreted as evidence of a Snowball event (*7*). These global-scale glaciations reflect the most marked climate changes in Earth’s known history. While the nature of Snowball climate dynamics (*3*, *8*) and the causes for the initiation and termination of Snowball episodes (*9*) have been topics of scientific interest, their actual causes still remain uncertain.

An early suggestion for the cause of Neoproterozoic Snowball episodes was the ∼6% lower solar insolation during that time (*10*). However, low insolation is not a satisfactory explanation, as reduced solar flux was likely compensated by higher atmospheric CO _2_ (*11*, *12*) and cannot explain the absence of glaciation for a billion years before the Sturtian event. Another suggested cause of Snowball events during the Neoproterozoic is the preponderance of continental landmasses in the low latitudes, which are expected to expose weatherable materials to the warm, wet tropics, as well as raise planetary albedo (*1*, *13*). While a low-latitude continental configuration has been shown to be favorable for transition to Snowball (*14*), little to no correlation has been identified between tropical land area and global temperatures over the past 400 Ma (*15*). It seems likely that the Neoproterozoic continental configuration provided favorable physical conditions for the initiation of a Snowball phase but was not a direct cause.

Additional suggestions for Snowball initiation appeal to variations in volcanic outgassing (*16*), changing continental configuration and composition (*17*–*19*), evolution of the biosphere (*20*, *21*), or the collapse of a methane greenhouse (*22*, *23*). While we do not dismiss these mechanisms, we note that any mechanism that calls for Snowball initiation via the lowering of atmospheric CO_2_ through biological or tectonic changes must explain how the proposed mechanism contends with and overcomes the stabilizing effect of the silicate weathering feedback (*24*, *25*), which is suggested to have maintained habitable conditions over much of Earth history. On the other hand, it is possible that weathering played an indirect role by cooling the climate and lowering the threshold for Snowball initiation in response to volcanism or impacts; the latter mechanism is the topic of this paper (*19*).

The final category of explanations for the initiation of Snowball Earth does not involve perturbations to the carbon cycle but calls for rapid changes in Earth’s albedo (*24*). For instance, one study has argued that the Sturtian Snowball may have been caused by injection of sulfate aerosols into the stratosphere during the emplacement of the Franklin large igneous province (LIP) (*26*). On the basis of a radiative model and mixed layer ocean, they argue the aerosol forcing arising from LIP volcanism could have tipped Earth into a Snowball. However, the latest geochronology indicates the emplacement of the Franklin LIP occurred 0.9 to 1.6 Ma before the initiation of the Sturtian Snowball phase, rendering volcanic initiation implausible (*19*). In addition, other studies show that the large heat capacity of the deep ocean, which rapidly overturns upon radiative cooling at the surface, requires continuous Toba-like explosive eruptions for decades to centuries to cool down to freezing temperature; this is unlikely to have occurred during the emplacement of LIPs (*27*, *28*).

Most relevant to the work presented here, two earlier studies argued based on an atmospheric energy balance model coupled to a two-dimensional ocean model, that the radiative effect of a large impact could transition the present-day climate to Snowball Earth (*29*, *30*). Furthermore, the possibility of impact-induced initiation or termination of Snowball Earth episodes has been hypothesized due to the coincidence (within uncertainty) of the Yarrabubba impact structure in Western Australia (2229 ± 5 Ma) with the youngest Paleoproterozoic glacial deposits (2225 ± 3 Ma) (*31*). The coincidence of Paleoproterozoic glacial deposits with an identified impact structure motivates us to revisit the idea of impact-induced Snowball Earth conditions, first proposed by Bendtsen and Bjerrum (*29*, *30*), with a state-of-the-art coupled atmosphere-ocean general circulation model (GCM) for a range of boundary conditions and impact scenarios.

The most salient example of the climatic effect of a large impact is the Chicxulub impact, which is associated with global impact winter and the K-Pg mass extinction (*32*–*35*). Proxy evidence and climate modeling studies of the K-Pg impact show an expansion of sea ice coverage and global cooling persisting for up to decades (*35*–*41*). In addition, other studies using coupled atmosphere-ocean GCMs, while not directly considering the role of a large impact, have found the Snowball transition time from the preindustrial (PI) climate upon complete elimination of solar insolation to be on the order of decades (*28*, *37*, *42*, *43*).

Here, we propose that one or more of the Paleoproterozoic or Neoproterozoic Snowballs could have been initiated by a large impact of extraterrestrial origin. We impose transient reductions in solar irradiance corresponding to the aftermath of a large impact for PI, Last Glacial Maximum (LGM; ∼21 ka), Cretaceous-like, and Neoproterozoic climate simulations. We note that our Cretaceous-like and Neoproterozoic simulations are subject to large uncertainties in paleogeography, global temperatures, and atmospheric composition; we therefore use the LGM and PI as reference climates with well-constrained global temperatures and well-reconstructed boundary conditions including land ice and pCO_2_. We find that an impact-induced Snowball Earth scenario requires a colder than PI background climate as well as a sufficiently strong radiative forcing. In contrast to earlier studies (*29*, *30*), we find that an impact with similar or greater radiative forcing compared to the K-Pg impact does not transition the PI climate into a Snowball yet easily initiates Snowball conditions given a colder, partially glaciated initial climate such as the LGM.

## RESULTS

We consider the simulated climate response following a Chicxulub-scale impact for several reference climates: the PI, LGM, a Cretaceous-like 4 × CO_2_ climate, and Neoproterozoic climates (summarized in Table 1). We use the K-Pg impact as an archetype of a large impact event that is known to have caused large climatic consequences. The 200-km-wide Chicxulub impact structure bears witness to the end of the Mesozoic era and the K-Pg mass extinction. The ∼10-km-diameter Chicxulub impactor struck the northern Yucatan peninsula, a site characterized by sulfur-rich marine anhydrites; upon impact, large amounts of sulfate aerosols and soot were injected into the stratosphere, leading to global cooling (*44*–*46*). The quantity of sulfur released by the Chicxulub impact event is orders of magnitude greater than by any known volcanic eruption (*47*).

**Table 1.** Summary of all model experiments. Boundary conditions, preimpact global mean surface temperature, preimpact sea ice coverage, and whether Snowball Earth initiation occurs in response to the 200-Gt impact scenario are shown. PI, preindustrial.

Experiment	CO_2_ (ppm)	Solar insolation	Preimpact surface temperature (°C)	Preimpact sea ice (%)	Snowball? (200-Gt SO_2_)
PI	284.7	100%	15.1	5.5	No
LGM	190	100%	8.3	10.4	Yes
4 × CO_2_	1138.8	100%	30.5	0.0	No
720 Ma (1500 ppm)	1500	94%	17.2	5.7	No
720 Ma (750 ppm)	750	94%	3.9	27.1	Yes

**Fig. 1.**
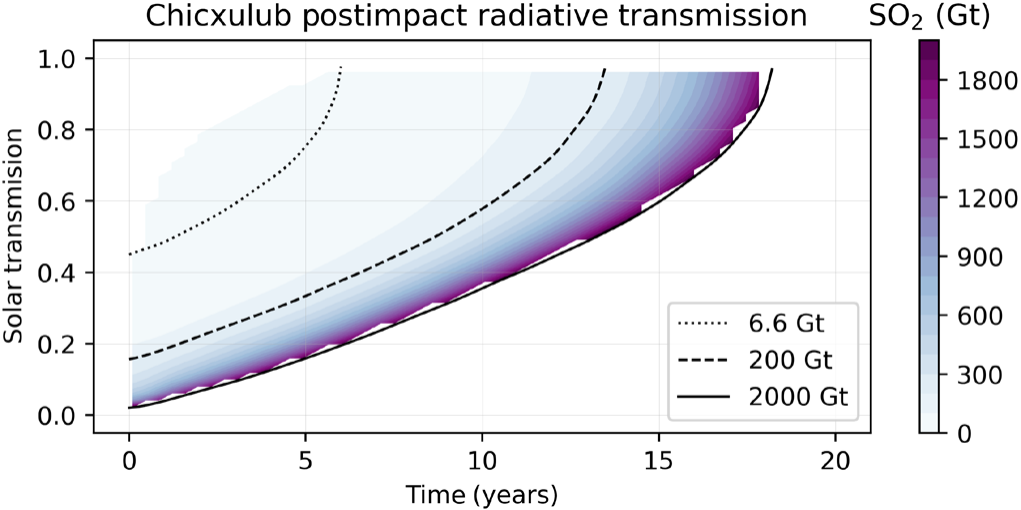
Atmospheric transmission at a wavelength of 500 nm as a function of time after impact. Figure adapted from calculations of Pope *et al.* (*46*). Postimpact radiative transmission relative to preimpact is shown for three scenarios in black curves, with transmission curves for other volumes of SO_2_ estimated through interpolation of the 6.6-, 200-, and 2000-Gt scenarios and shown in colored contours.

The short-term cooling resulting from different scenarios of sulfate and greenhouse gas release has been evaluated in prior studies (*44*–*46*), indicating decreases in solar transmission at the surface persisting for up to a few decades. Here, we adopt the radiative calculations of Pope *et al.* (*46*) for three scenarios of stratospheric SO_2_ injection: 6.6, 200, and 2000 Gt. Following Pope *et al.*, we adopt 200 Gt as a conservative yet plausible injection of sulfates, but we also simulate the climate response to 6.6- and 2000-Gt scenarios. The radiative forcing of these three scenarios is shown in Fig. 1.

We proceed to prescribe abrupt, transient reductions in insolation following the curves in Fig. 1 to a range of climate simulations using the Community Earth System Model version 1 (CESM1). CESM is a state-of-the-art atmosphere-ocean climate model developed by the National Center for Atmospheric Research. Our PI simulation follows a standard setup, whereas the LGM simulations use 21-ka boundary conditions including continental ice sheets and an atmospheric CO_2_ of 190 parts per million (ppm). Our Cretaceous-like simulation has been run to quasi-equilibrium at 4× PI atmospheric CO_2_ (1138.8 ppm) and, for simplicity, uses PI boundary conditions. Our Neoproterozoic simulations use 720-Ma paleogeography, but due to uncertain atmospheric CO_2_, we test two scenarios: one with a background atmospheric CO_2_ concentration of 1500 ppm and another with 750 ppm. These two simulations are run with solar insolation set to 94% of present day and result in equilibrium mean ocean temperatures comparable to the PI and LGM simulations, respectively. Further details are provided in Materials and Methods.

We first benchmark our modeling framework by evaluating the climate response of our Cretaceous-like 4 × CO_2_ simulation to the three radiative forcing scenarios (fig. S1). The transient decreases in global-mean surface temperature and transient top-of-atmosphere radiative imbalance are in general agreement with the results of a number of other climate modeling studies of the K-Pg impact (*35*–*41*), which gives us confidence that our imposed radiative forcing scenarios are realistic. We proceed to investigate the hypothetical effects of these impact scenarios applied to PI, LGM, and Neoproterozoic climates.

**Fig. 2.**
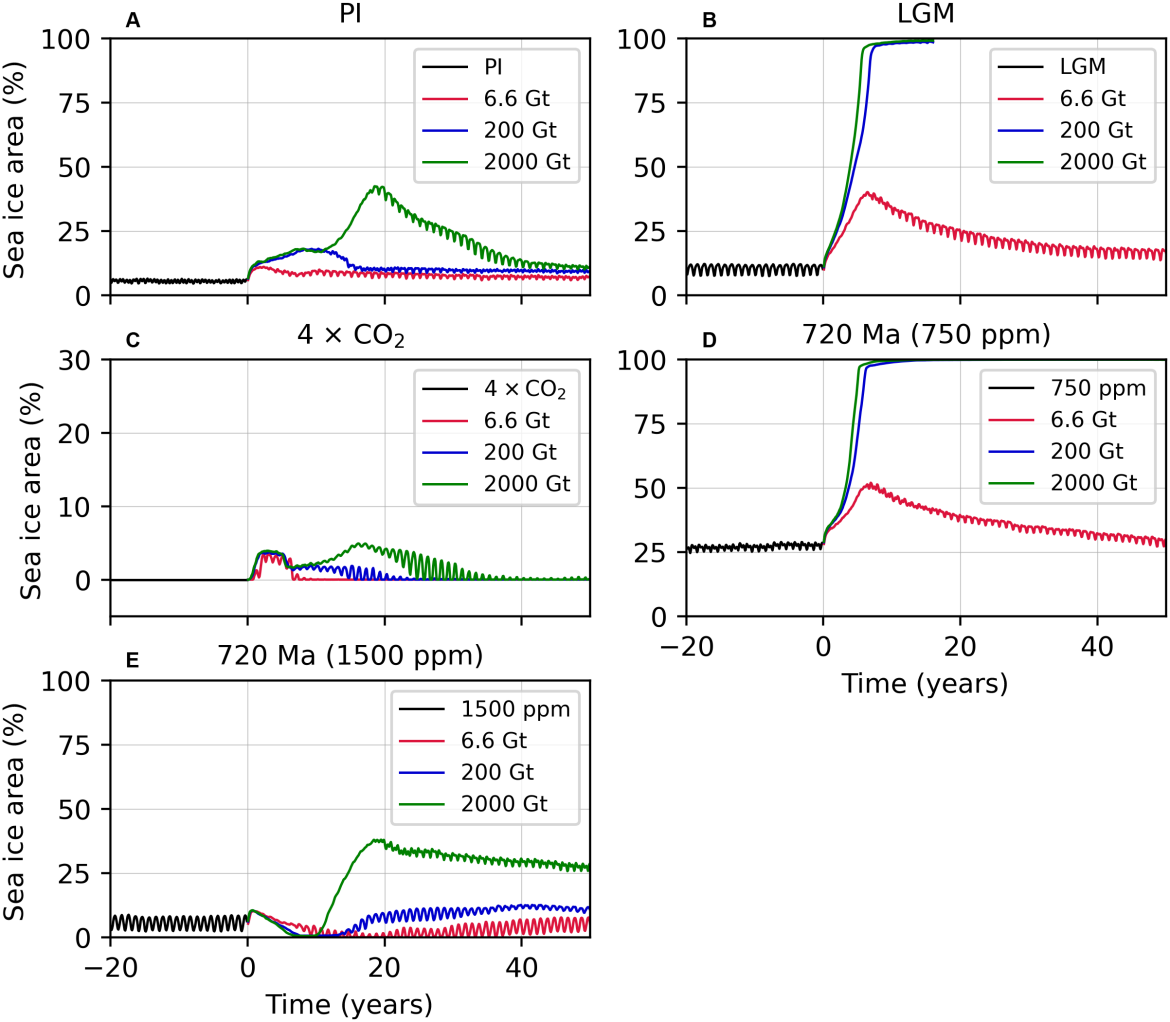
Response of sea ice coverage to radiative forcing under 6.6-, 200-, and 2000-Gt sulfate aerosol injection scenarios. (**A**) Sea ice coverage in the preindustrial (PI) simulations. (**B**) As in (A) but for the LGM simulations. (**C**) As in (A) but for the 4 × CO_2_ simulations. (**D**) As in (A) but for the 720-Ma (750-ppm) simulations. (**E**) As in (A) but for the 720-Ma (1500-ppm) simulations. Snowball Earth (defined here as 97% global sea ice coverage) is achieved on the ninth year for the 200-Gt scenario and the seventh year for the 2000-Gt scenario applied to the LGM simulation. Snowball Earth is achieved on the eighth and sixth years, respectively for the 720-Ma (750-ppm) simulation.

The response of global sea ice coverage to the three radiative forcing scenarios is shown in Fig. 2. For the 200-Gt scenario, which we take as a plausible Chicxulub-like scenario, an increase in sea ice coverage is seen in the PI climate response, reaching a maximum of 20% after around one decade. The response to the 6.6-Gt scenario is weaker, whereas the 2000-Gt scenario shows a rise to just over 40% sea ice coverage at around year 20 that subsequently reverses. Therefore, in contrast to Bendtsen and Bjerrum (*29*, *30*), we find it unlikely that a Chicxulub-scale impact could transition the PI climate to Snowball Earth conditions.

The response of the LGM climate to the prescribed radiative forcing, however, differs radically from the PI climate. Following the 200-Gt scenario, the LGM climate instead shows a rapid transition to 95% ice coverage after only 8 years and achieves 97% sea ice coverage by the ninth year. The 6.6-Gt scenario, which is not sufficient to initiate Snowball conditions, still leads to a remarkable increase in sea ice coverage of up to 40% by the seventh year, which subsequently reverses. This suggests that a runaway ice-albedo feedback in the aftermath of a large impact is highly sensitive to the initial state of the climate, particularly the ocean, as the cold LGM ocean waters are far more susceptible to sea ice formation than those of preindustrial (and warmer) climates. The response of sea ice coverage for this simulation is also shown in movie S1.

The response of the 4 × CO_2_ climate to the various impact scenarios is more muted, showing a transient increase in sea ice coverage to a few percent that subsequently reverses (Fig. 2B). The two Neoproterozoic simulations with 720-Ma paleogeography show a comparable response to the LGM and PI simulations, with the colder 750-ppm scenario entering Snowball Earth conditions whereas the warmer 1500-ppm scenario does not. This demonstrates that the impact-induced Snowball Earth mechanism is sensitive to ocean temperatures but not to detailed aspects of paleogeography. The response of sea ice coverage for the 720-Ma (750-ppm) simulation is shown in movie S2.

**Fig. 3.**
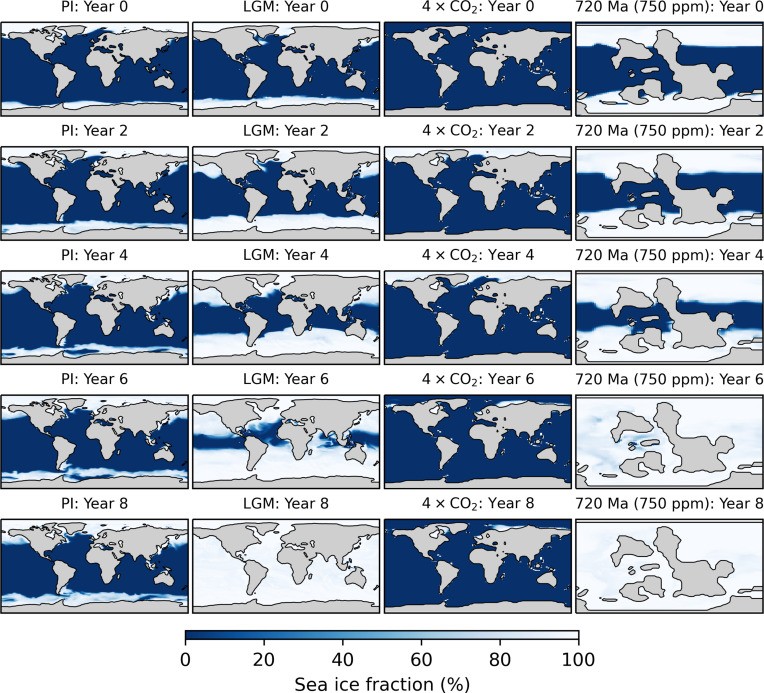
Snapshots of sea ice coverage during the decade following the 200-Gt radiative forcing scenario for selected experiments. From left to right, columns show the sea ice response of the preindustrial (PI), LGM, 4 × CO_2_, and 720-Ma (750-ppm) simulations, respectively.

The response of global sea ice coverage a few years postimpact (under the 200-Gt scenario) is shown in Fig. 3. While the PI climate exhibits modest expansion of sea ice over the North Atlantic, North Pacific, and Southern Ocean, these changes are transient and reverse after one decade. On the other hand, sea ice grows very rapidly for the LGM simulation, with the sea ice margin reaching into the mid-latitudes by the fourth year and into the deep tropics by the sixth year. By this point, the sea ice margin has exceeded the critical threshold for unstoppable glaciation, and by the ninth year, global ice coverage has exceeded 97% and the planet has entered a hard Snowball Earth. The 4 × CO_2_ simulation shows a modest growth of sea ice in the Northern Hemisphere that quickly reverses, while the 720-Ma (750-ppm) simulation rapidly enters Snowball conditions, similar to the response of the LGM simulation. Figures S2 and S3 show the response of sea ice thickness for the LGM and 720-Ma (750-ppm) simulations, indicating that sea ice of over 10-m depth has formed along the equator by the end of the simulations.

Previous studies have suggested that it is important to account for ocean circulation and deep ocean temperatures when considering the climate response to changes in insolation and planetary albedo (*27*). Figures S4 to S8 show zonal-averaged ocean temperatures as a function of latitude and depth for the 200-Gt scenario applied to LGM, PI, Neoproterozoic, and 4 × CO_2_ climates, respectively. Figure S4 confirms that the LGM ocean has almost entirely cooled down to freezing temperature before Snowball initiation. The PI ocean, which by contrast does not enter Snowball conditions, retains heat within the deep ocean (fig. S5). Similarly, the 720-Ma (1500-ppm) simulation retains heat in the deep ocean, whereas the 720-Ma (750-ppm) simulation, which enters Snowball conditions, has cooled down to freezing temperature (figs. S6 and S7). The 4 × CO_2_ simulation, as expected, is far too warm to allow Snowball initiation and retains considerable ocean heat content (fig. S8).

The global meridional overturning circulation (GMOC) for the LGM, PI, Neoproterozoic, and 4 × CO_2_ simulations exhibits extremely vigorous and deep overturning in response to postimpact radiative cooling at the surface for all simulations (figs. S9 to S13). Figure S14 shows time series of GMOC strength in all experiments, with GMOC transiently reaching a peak of over 300 Sv for the PI ocean and around 100 Sv for the LGM ocean under the 200-Gt scenario. Similar invigoration of the overturning circulation is seen in the 4 × CO_2_ and 720-Ma simulations.

**Fig. 4.**
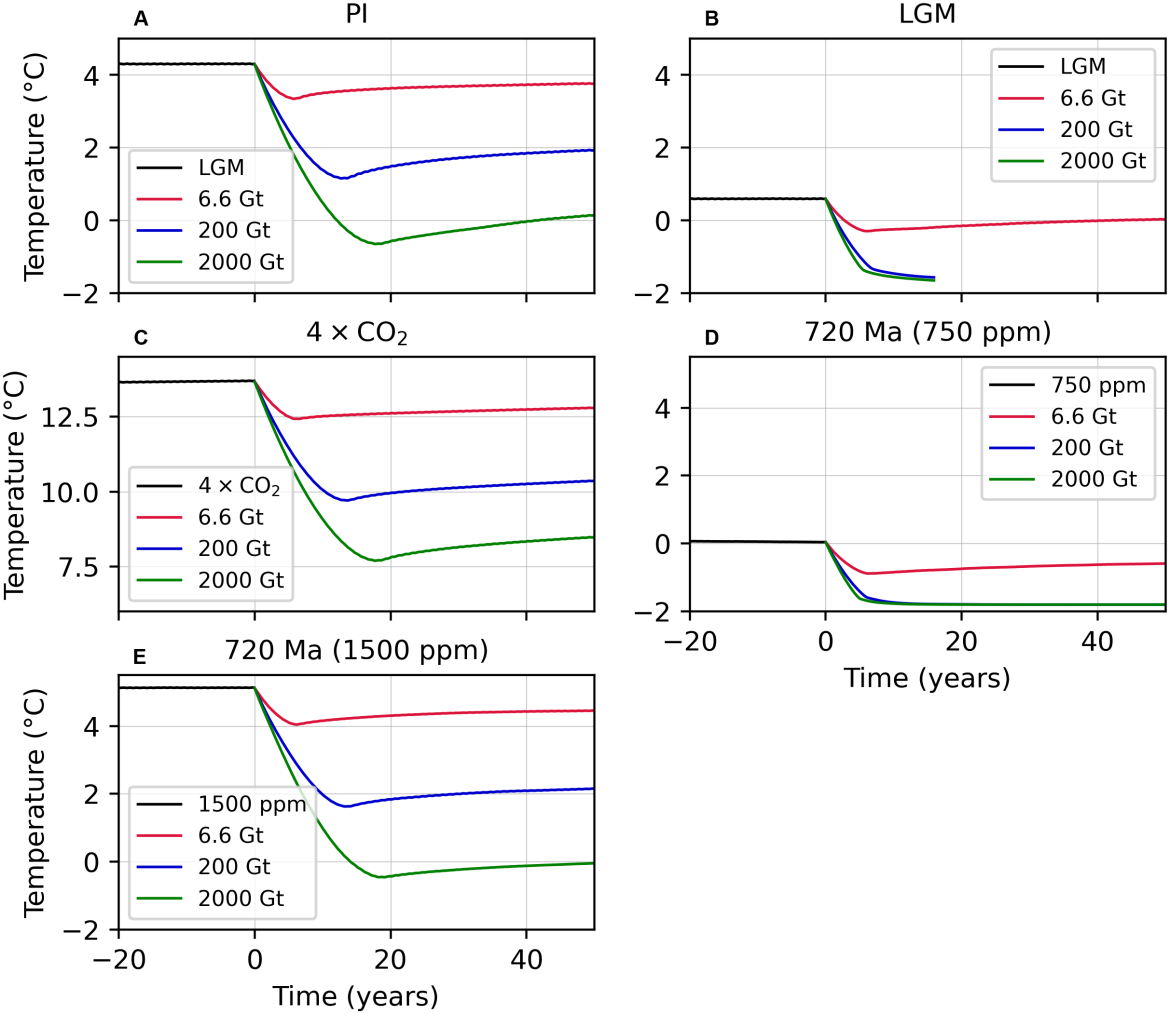
Global mean ocean temperature (averaged over all depths) for 6.6-, 200-, and 2000-Gt sulfate aerosol injection scenarios. (**A**) Ocean temperature response for the PI simulations. (**B**) As in (A) but for the LGM simulations. (**C**) As in (A) but for the 4 × CO_2_ simulations. (**D**) As in (A) but for the 720-Ma (750-ppm) simulations. (**E**) As in (A) but for the 720-Ma (1500-ppm) simulations.

Given rapid postimpact overturning of the ocean and hence the importance of deep ocean temperatures for Snowball initiation, we plot the depth-averaged global-mean ocean temperature for all model experiments in Fig. 4. None of the model experiments for the PI, 4 × CO_2_, or 720-Ma (1500-ppm) climates show global ocean temperatures reaching the freezing point of sea water (−1.8°C). On the other hand, the 200- and 2000-Gt scenarios for the LGM and 720-Ma (750-ppm) climates, which do lead to Snowball initiation, indeed feature mean ocean temperatures that reach approximately −1.8°C, confirming that the entire water column must cool to near-freezing temperatures before Snowball transition can occur (*28*).

We also tested the effect of a large injection of CO_2_ that is expected to occur following a large impact. We tried abruptly raising atmospheric CO_2_ by 1000 ppm for the 200-Gt scenario applied to the LGM climate. This had virtually no effect, as the changes in shortwave forcing dominate over the relatively small longwave radiative forcing of the CO_2_ injection. This is consistent with the negligible short-term effect of the release of greenhouse gases following a large impact (*45*); the responses of sea ice area and mean ocean temperatures for this experiment are shown in fig. S15.

## DISCUSSION

In this study, we used coupled ocean-atmosphere climate simulations to show that impact winter, following a large impact comparable in size to the Chicxulub event, could have transitioned a cold background climate (such as the LGM or a Neoproterozoic climate with atmospheric CO_2_ concentration of ∼750 ppm) to Snowball Earth. Unexpectedly, the concept of impact-induced Snowball initiation has received limited attention in the literature (*29*, *30*). In addition, we have shown that, by contrast, the PI, Cretaceous, and 720-Ma (1500-ppm) climates do not readily transition to Snowball conditions, even for a large impact where 2000 Gt of SO_2_ is released into the stratosphere.

While we are not aware of direct geological evidence that indicates that Paleoproterozoic or Neoproterozoic Snowballs were caused by an impact event, it is possible that a hypothetical impact crater could have been subducted or eroded away or has not yet been discovered. Furthermore, had an impact occurred in the deep ocean, it is unlikely for a crater to remain, as there is no ocean floor of that age preserved. Although we did not directly address the case of an impact in the deep ocean, we believe that such a scenario is unlikely to result in the necessary cooling for Snowball initiation, as an impact into oceanic crust has been argued to result in only modest cooling (*48*). The radiative effects of the large release of water vapor in response to an oceanic impact would be short-lived and would likely have a greenhouse warming effect. We note that the possible role of large impacts in the deglaciation of Snowball periods has also been proposed, although this has yet to be investigated from a climate perspective (*31*, *49*).

Given the limited preservation of impact structures on the Earth’s surface, which are subject not only to erosion and/or being covered by postimpact rock units, and the fact that any oceanic impacts would not leave a structural trace after such a long time, it is probably not unexpected that no impact structure of the relevant age has yet been identified. Even in the case of the Chicxulub structure, 10 times younger, it took many years of intense efforts after the discovery of impact evidence to find the (covered) impact structure (*50*). Thus the most promising way to detect the remnants of any possible impact event would be by chemically and petrographically identifying a distal ejecta layer, similar to any of the early Proterozoic ejecta layers (*51*). It should be noted that no such deposit has been identified so far, but there has also not been any dedicated search.

We can estimate, to first order, the probability of impact-induced Snowball episodes. We assume that an object of greater than 5-km diameter is sufficient to induce Snowball conditions given a suitable background climate and impact site; an object of this size or larger strikes the Earth every ∼40 Ma (*52*–*54*). A recent study has suggested that 13% of the Earth’s surface is covered by hydrocarbon-rich materials that would lead to marked global cooling upon large impact (*48*). We estimate 50 Ma of the Phanerozoic (538.8 Ma to present) such as during the Late Ordovician (448 to 440.5 Ma), Permo-Carboniferous (335 to 295 Ma), and the Pleistocene (2.6 Ma to 10.7 ka) was in an icehouse climate, defined here as land ice exceeding the 60 latitude threshold based on the reconstruction of Macdonald *et al.* (*55*).

If we assume the Phanerozoic temperature distribution and sulfate-rich surface area fraction were comparable during earlier periods, we estimate an impact-induced Snowball every ∼3.3 Ga. Using the binomial distribution for independent events, this leads to a ∼53% probability that an impact-induced Snowball has occurred at least once since 2.5 Ga, a ∼18% chance it has happened at least twice, and a ∼4% chance that it has happened at least three times. However, the long-term temperature distribution and area fraction of sulfate-rich sediments before the Phanerozoic are highly uncertain. The relative scarcity of marine sulfate evaporites during the middle Proterozoic, for instance, may help explain the absence of global glaciations during this period (*56*).

We note that an impact-induced Snowball would differ from other mechanisms for Snowball initiation in several key ways, some which may be geologically testable. For instance, while most suggested mechanisms for Snowball initiation call for a lowering of atmospheric CO_2_ through biological or tectonic changes, a large impact is likely to have caused an abrupt surge in atmospheric CO_2_ through the the vaporization of sedimentary materials. Furthermore, the advance of the sea ice margin from the high latitudes into the tropics could have been extremely abrupt under an impact-induced scenario. As we have shown here, an impact-induced transition from the LGM to Snowball Earth occurs within one decade, compared to the long timescales of weathering-induced cooling which imply a gradual advance of the sea ice margin toward the critical latitude (of about 30 to 40) over a timescale of millions of years. This implies that glacial sediments over mid-latitude oceans are less likely for an impact-induced Snowball.

In conclusion, we have shown, on the basis of climate modeling, that the initiation of Snowball Earth by an asteroid impact is a plausible mechanism given a cold background climate. Although the concept of an impact-induced Snowball remains theoretical, we note that suggestions of a cold Tonian climate ∼63 to 13 Ma before the onset of the Sturtian Snowball (*57*, *58*) lend some credibility to the ideas presented here. However, evidence of cold Tonian conditions is controversial (*5*, *59*), and climate conditions just before the onset of the Sturtian event (∼717 Ma) remain uncertain; better constraints are needed to evaluate the plausibility of volcanic and impact-triggered initiation mechanisms. Furthermore, our finding that had a Chicxulub-sized impact occurred during the LGM, a Snowball Earth would likely have ensued, suggests that the Pleistocene ice ages may have been on the brink of global-scale glaciation in response to strong stochastic forcing. As roughly half of the 2.6 Ma Pleistocene was characterized by extensive glaciation, and ∼13% of Earth’s surface area is volatile rich, this implies an approximately 1/250 chance of Snowball initiation during the Pleistocene alone, which represents only one glacial period out of many in Earth’s geological history.

## MATERIALS AND METHODS

We simulate the climate response to impact winter for a variety of background climates using the CESM1.2.2 climate model in a fully coupled atmosphere-ocean configuration. CESM uses the Community Atmosphere Model version 5 (CAM5) (*60*) as its atmospheric component and the Parallel Ocean Program version 2 (*61*) as its ocean component.

For our LGM and PI simulations, we use a medium horizontal resolution of 2 in the atmosphere and 1 in the ocean. The LGM simulation follows the setup of a previous study that used PMIP4 LGM boundary conditions, including an atmospheric CO_2_ concentration of 190 ppm, land ice from the ICE-6G reconstruction (*62*), and a modified land-sea mask corresponding to lower LGM sea level (*63*). The PI is first spun up for 1100 years from the default ocean initial condition, while the LGM simulation is spun up for 1100 years with the ocean state starting from the end of an earlier CCSM4 simulation of the LGM that was integrated for over 2400 years. Both the PI and LGM show near-zero top-of-atmosphere energy imbalances by the end of the spin-up period with stable deep ocean temperatures.

The weaker constraints on atmospheric CO_2_ and paleogeography justify the use of a coarser resolution of ∼4 in the atmosphere and ∼3 in the ocean for our Cretaceous-like and Neoproterozoic simulations. The Neoproterozoic simulation used 720-Ma paleogeography from the reconstruction of Merdith *et al.* (*64*). Because of limited constraints on topography and bathymetry, we used a simplified 100-m elevation for continental land and a uniform 3700-m bathymetry for the ocean with no land ice. Solar insolation was set to 94% of present-day, and we evaluated background CO_2_ concentrations of 750 and 1500 ppm, a range broadly consistent with estimates by Mills *et al.* (*12*). The Neoproterozoic simulations were spun up from horizontally uniform ocean salinity and temperatures profiles for 2000 years until ocean temperatures stabilized. Land surface was set to bare ground, and the orbit was set to 23.5° obliquity with zero eccentricity.

To benchmark our modeling framework, we also simulated the climate response to a large impact for a 1138.8 ppm (4× PI CO_2_) climate, intended as an analog for the warm upper Cretaceous climate. Ocean temperatures and salinity were initialized from a 6000-year-long simulation with an atmospheric CO_2_ concentration of 1138.8 ppm using CESM1.0.4 and further integrated in CESM1.2.2 (CAM5) for 1200 years. While we discuss the relevance of our results to Paleoproterozoic glaciations, we did not explicitly simulate Paleoproterozoic climates and their response to large impacts due to poorly constrained continental configuration, atmospheric composition, and other boundary conditions for this period.
