# Supplementary material for: Impact-induced initiation of Snowball Earth: A model study
Source: Sci Adv. 2024 Feb 9;10(6):eadk5489. doi: 10.1126/sciadv.adk5489 (PMC10857373; doi:10.1126/sciadv.adk5489)
Supplement: Supplementary file 1 — Figs. S1 to S15 Legends for movies S1 and S2 [file sciadv.adk5489_sm.pdf]

Science Advances
AAAS

# Supplementary Materials for

## Impact-induced initiation of Snowball Earth: A model study

Minmin Fu *et al.*

Corresponding author: Minmin Fu, minmin.fu@yale.edu

*Sci. Adv.* **10**, eadk5489 (2024)
DOI: 10.1126/sciadv.adk5489

**The PDF file includes:**

Figs. S1 to S15
Legends for movies S1 and S2

**Other Supplementary Material for this manuscript includes the following:**

Movies S1 and S2

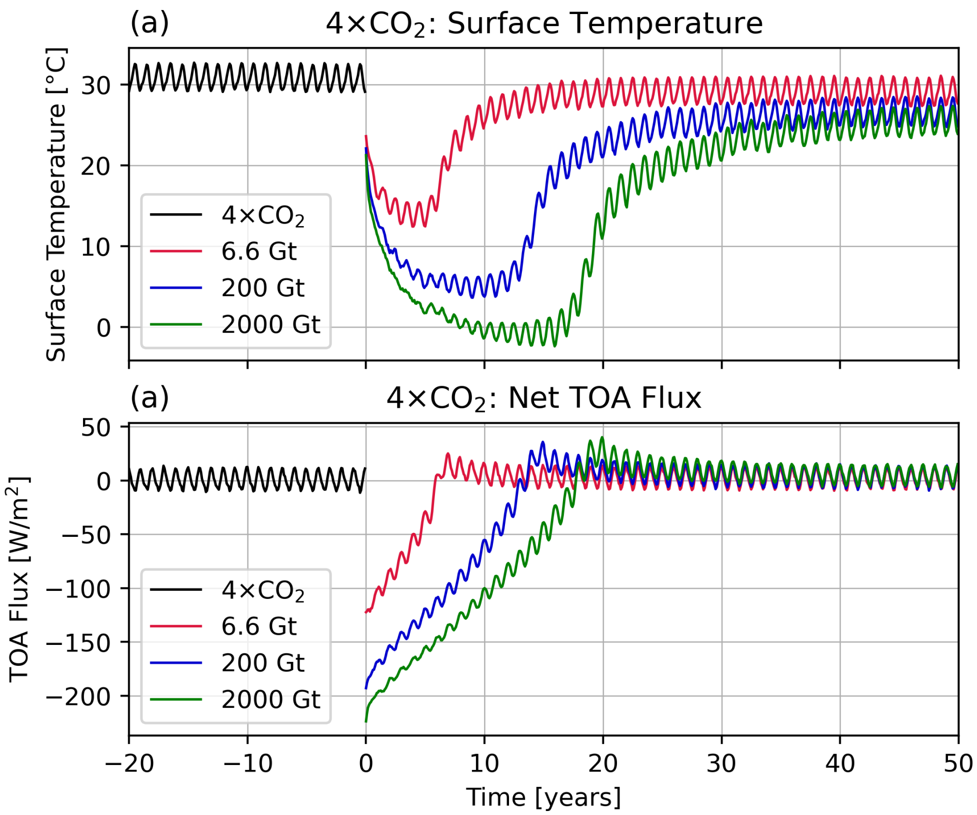


Figure S1: **Response of surface temperature and top-of-atmosphere (TOA) energy imbalance for the 4$\times$$CO_2$ simulations in response to 6.6, 200, and 2000 Gt sulfate aerosol injection scenarios.** (a) Global-mean surface temperature response. (b) Net TOA energy imbalance.

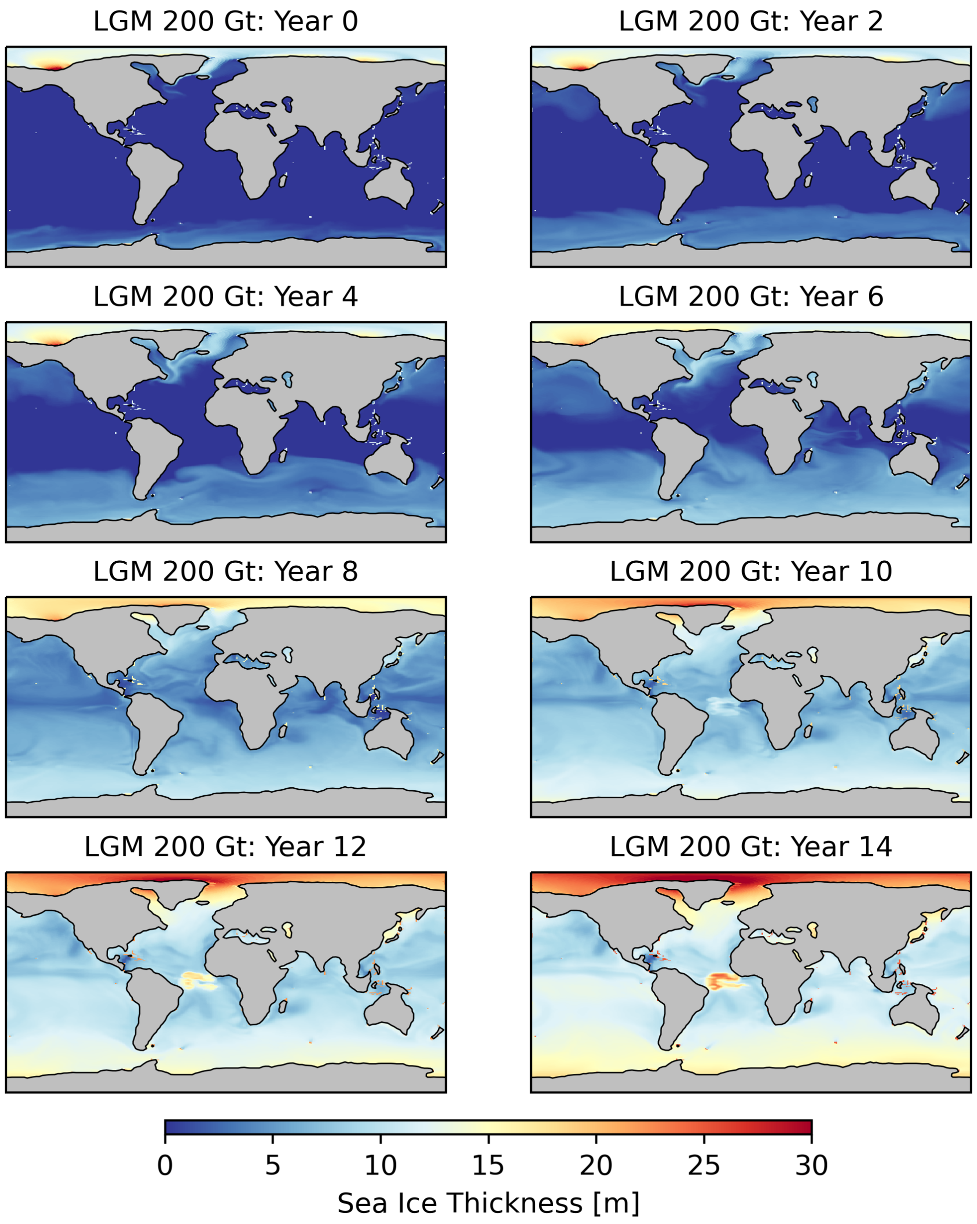


Figure S2: **Snapshots of sea ice thickness following the 200 Gt radiative forcing scenario applied to the LGM climate.**

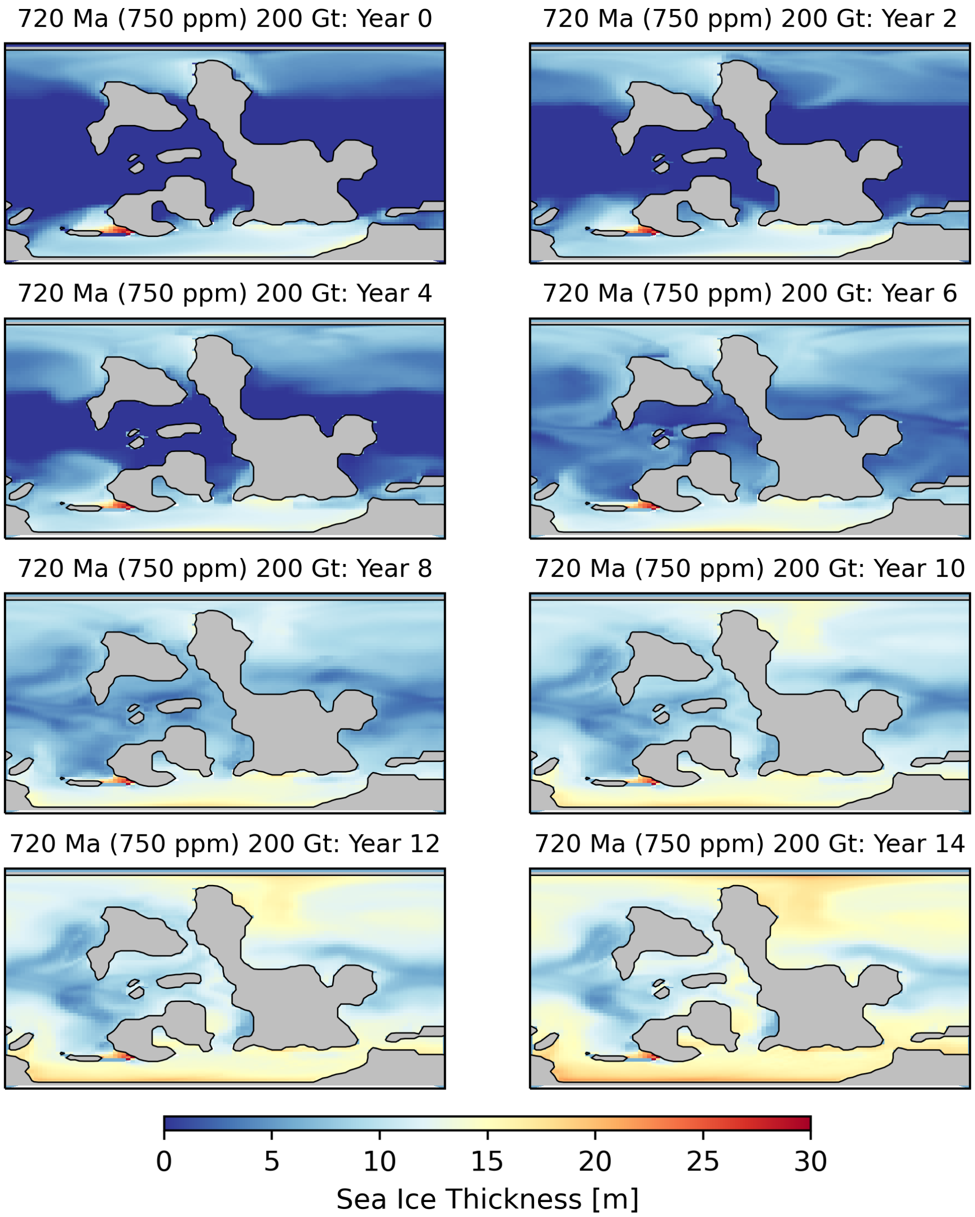


Figure S3: **Snapshots of sea ice thickness following the 200 Gt radiative forcing scenario applied to the 720 Ma (750 ppm) climate.**

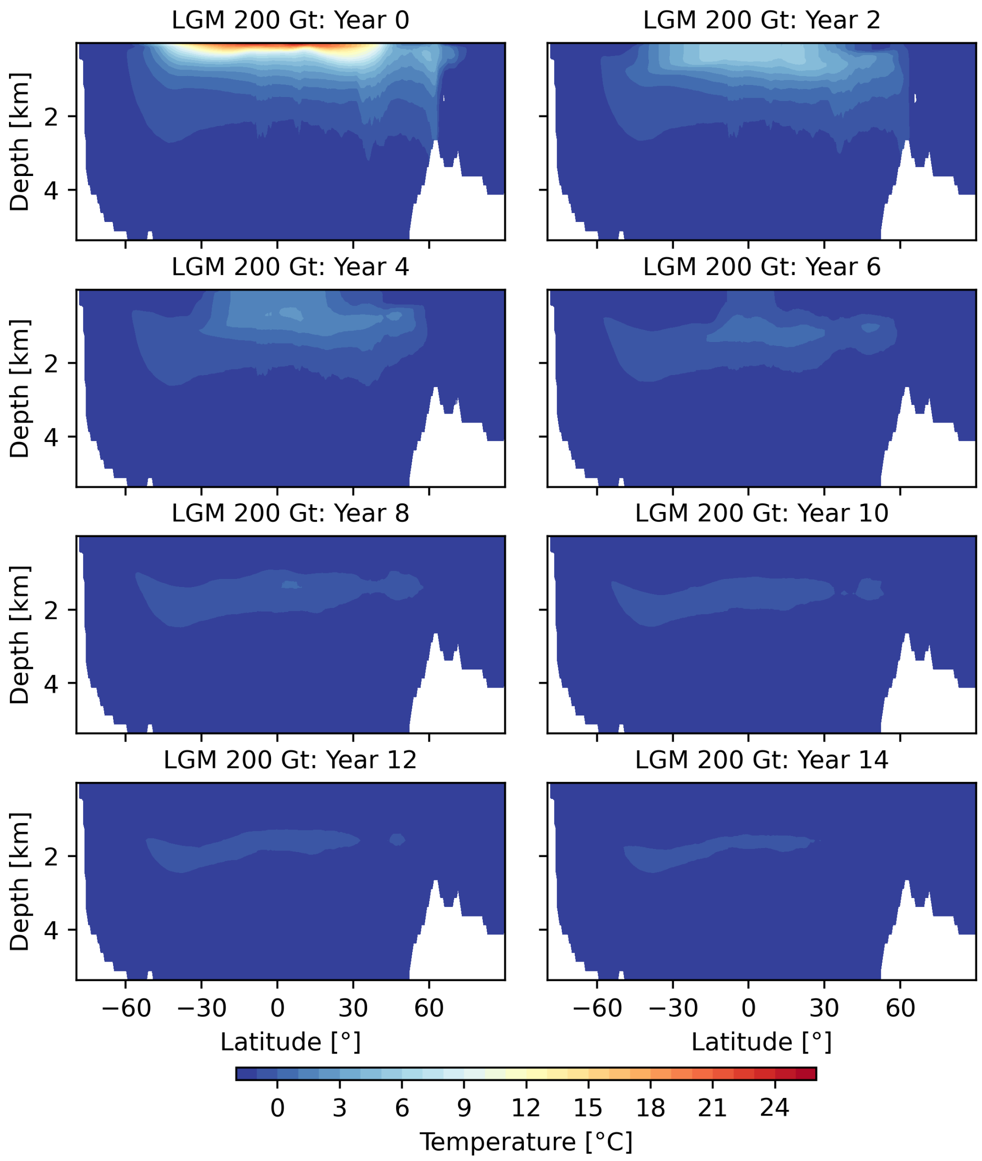


Figure S4: **Snapshots of zonal-mean ocean temperatures as a function of latitude and depth, following the 200 Gt radiative forcing scenario applied to the LGM climate.**

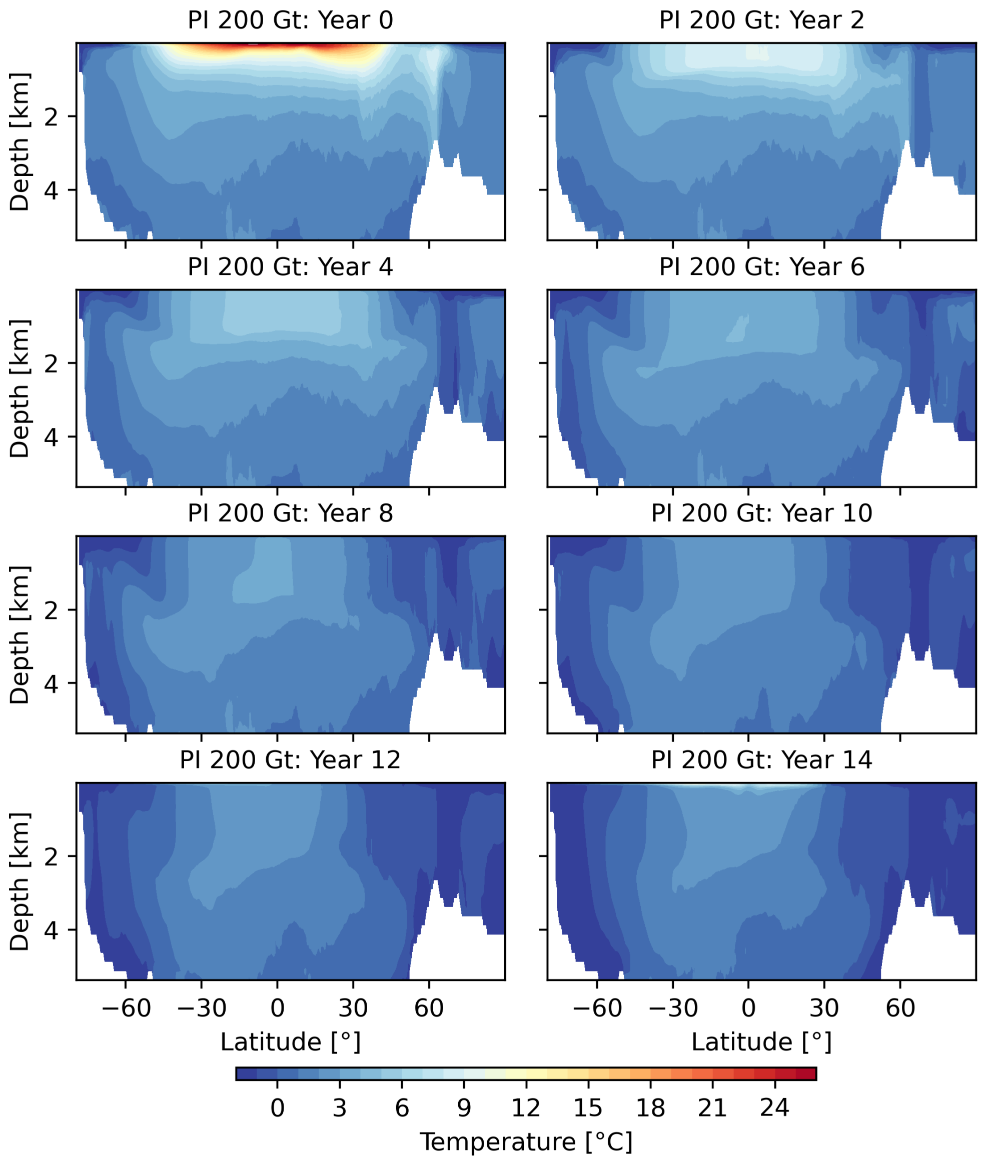


Figure S5: **Snapshots of zonal-mean ocean temperatures as a function of latitude and depth, following the 200 Gt radiative forcing scenario applied to the preindustrial climate.**

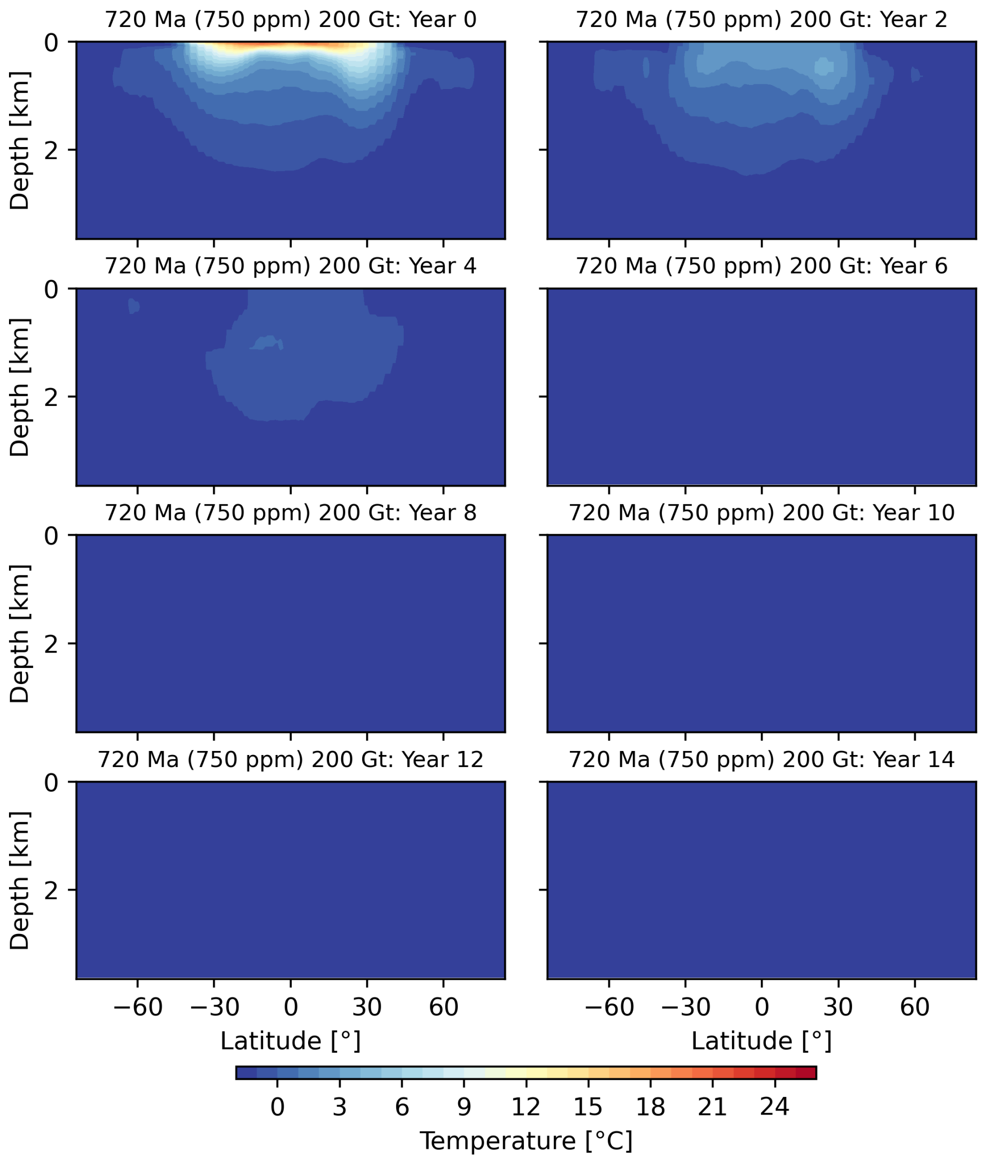


Figure S6: **Snapshots of zonal-mean ocean temperatures as a function of latitude and depth, following the 200 Gt radiative forcing scenario applied to the 720 Ma (750 ppm) climate.**

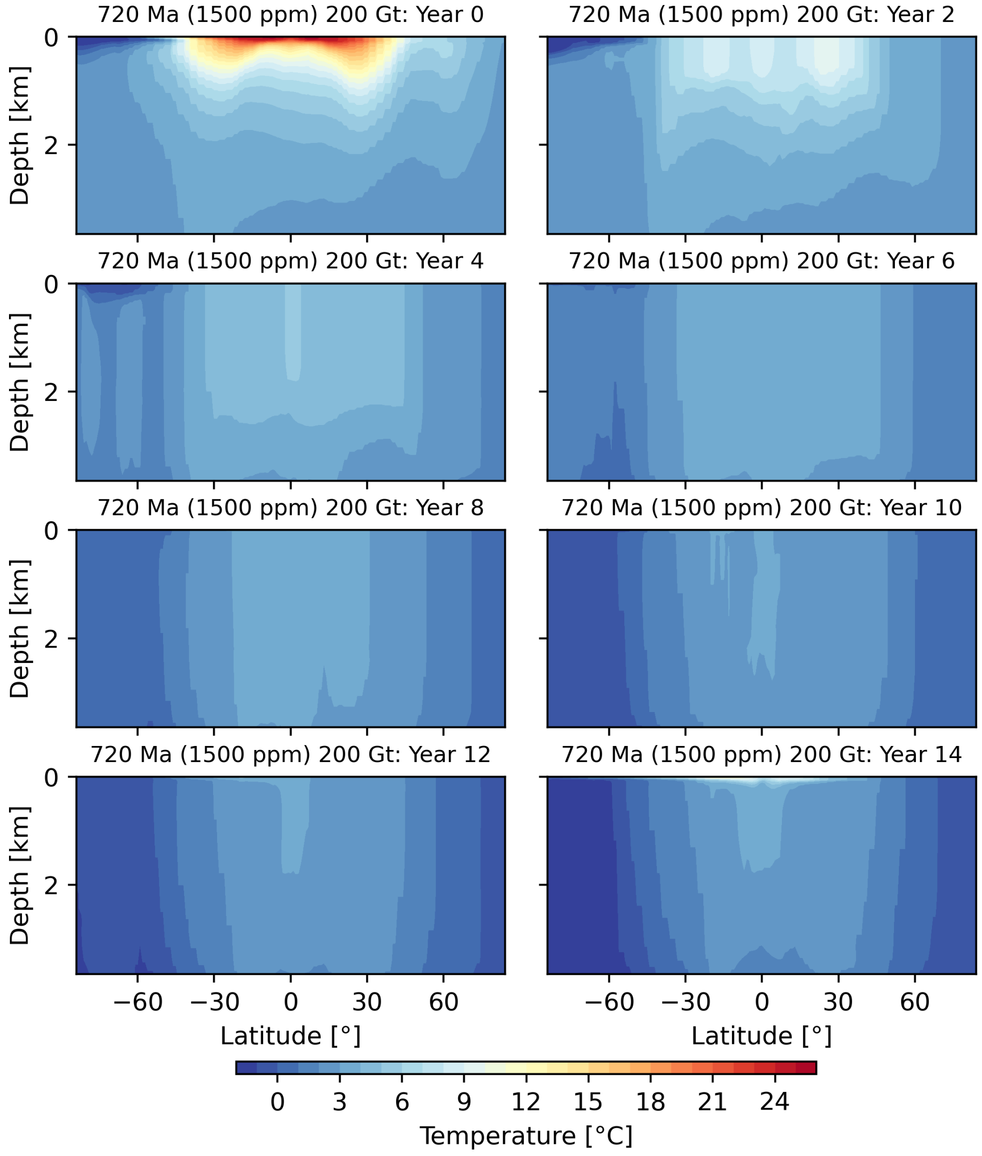


Figure S7: **Snapshots of zonal-mean ocean temperatures as a function of latitude and depth, following the 200 Gt radiative forcing scenario applied to the 720 Ma (1500 ppm) climate.**

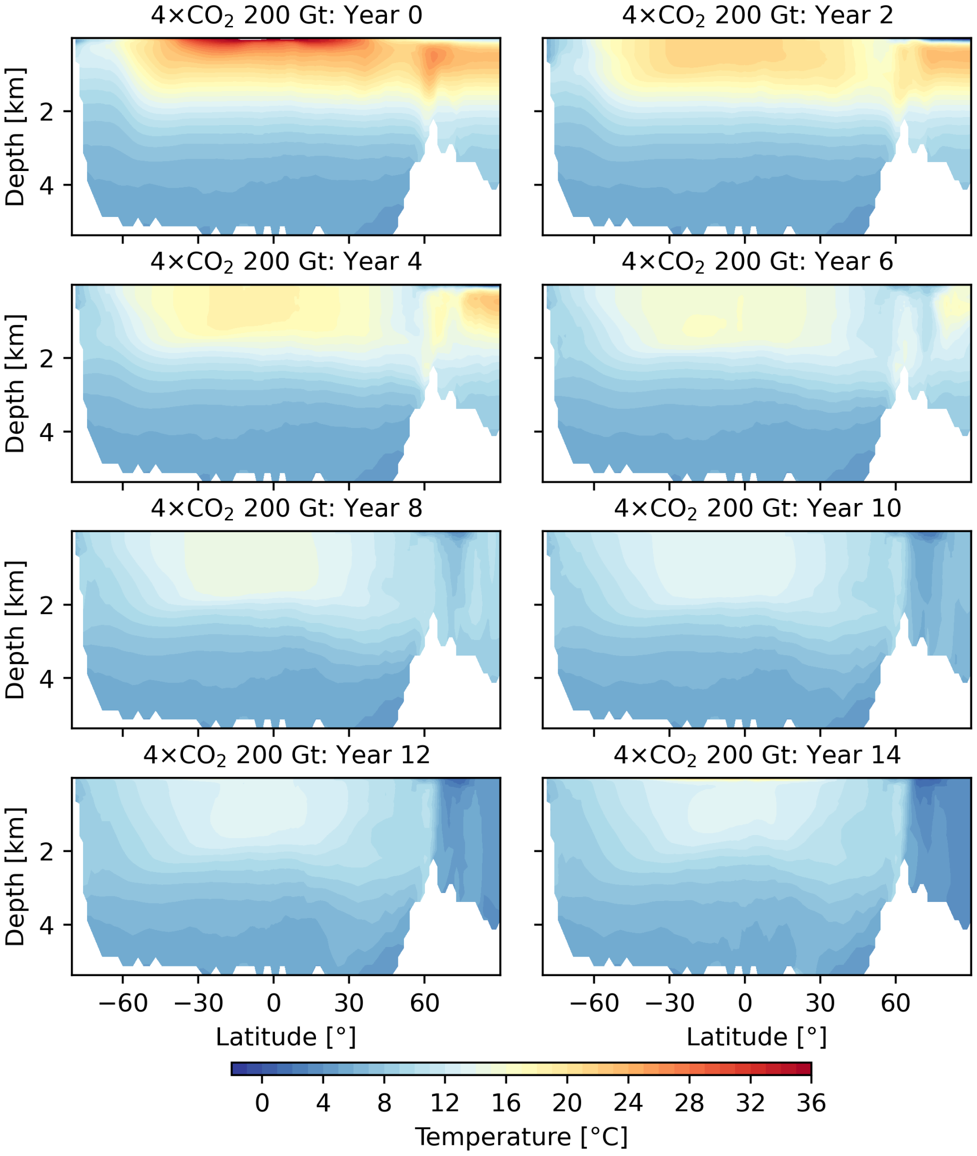


Figure S8: **Snapshots of zonal-mean ocean temperatures as a function of latitude and depth, following the 200 Gt radiative forcing scenario applied to the 4×$CO_2$ climate.**

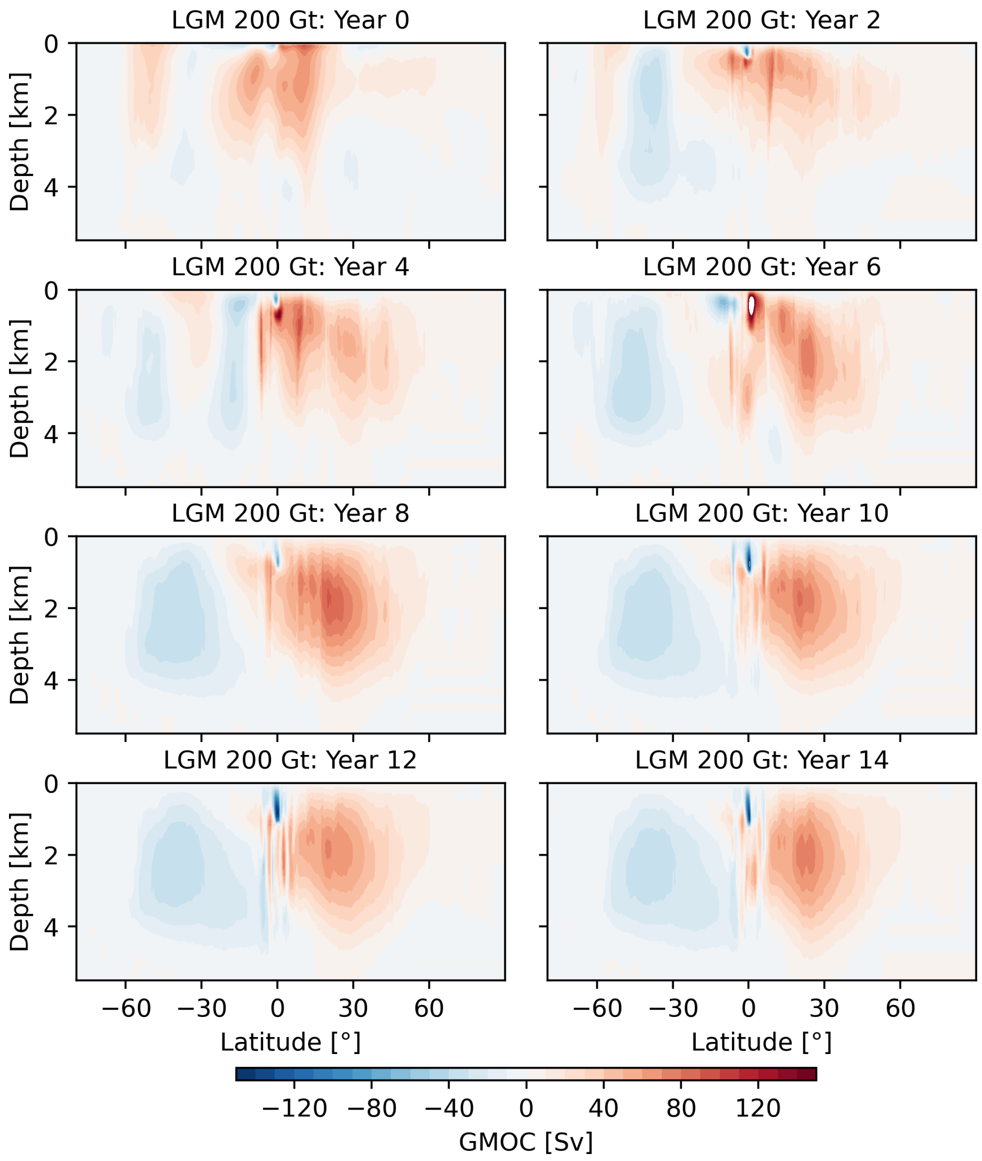


Figure S9: **Eulerian streamfunction for the global meridional overturning circulation, shown as a function of latitude and depth, following the 200 Gt radiative forcing scenario applied to the LGM climate.**

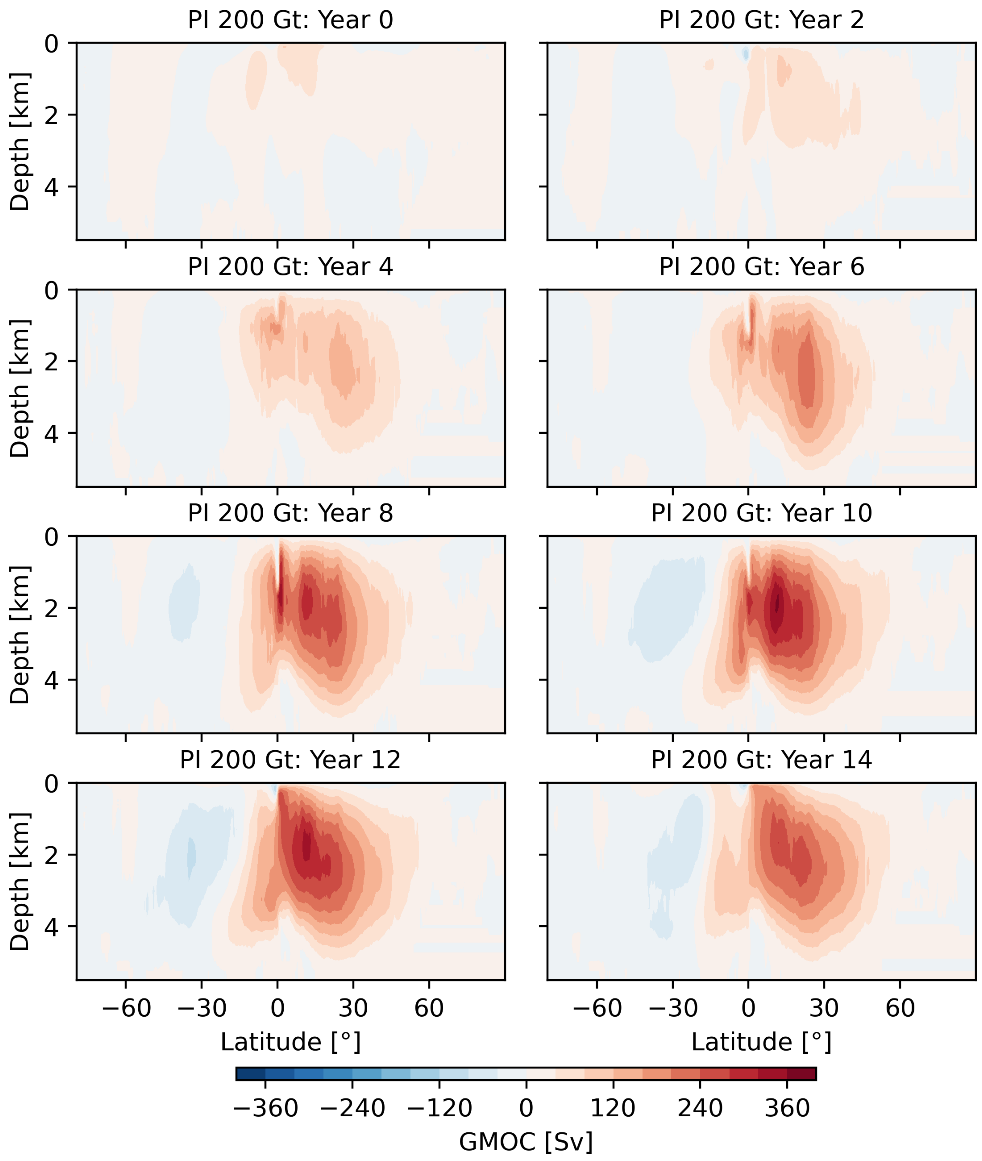


Figure S10: **Eulerian streamfunction for the global meridional overturning circulation, shown as a function of latitude and depth, following the 200 Gt radiative forcing scenario applied to the preindustrial climate.**

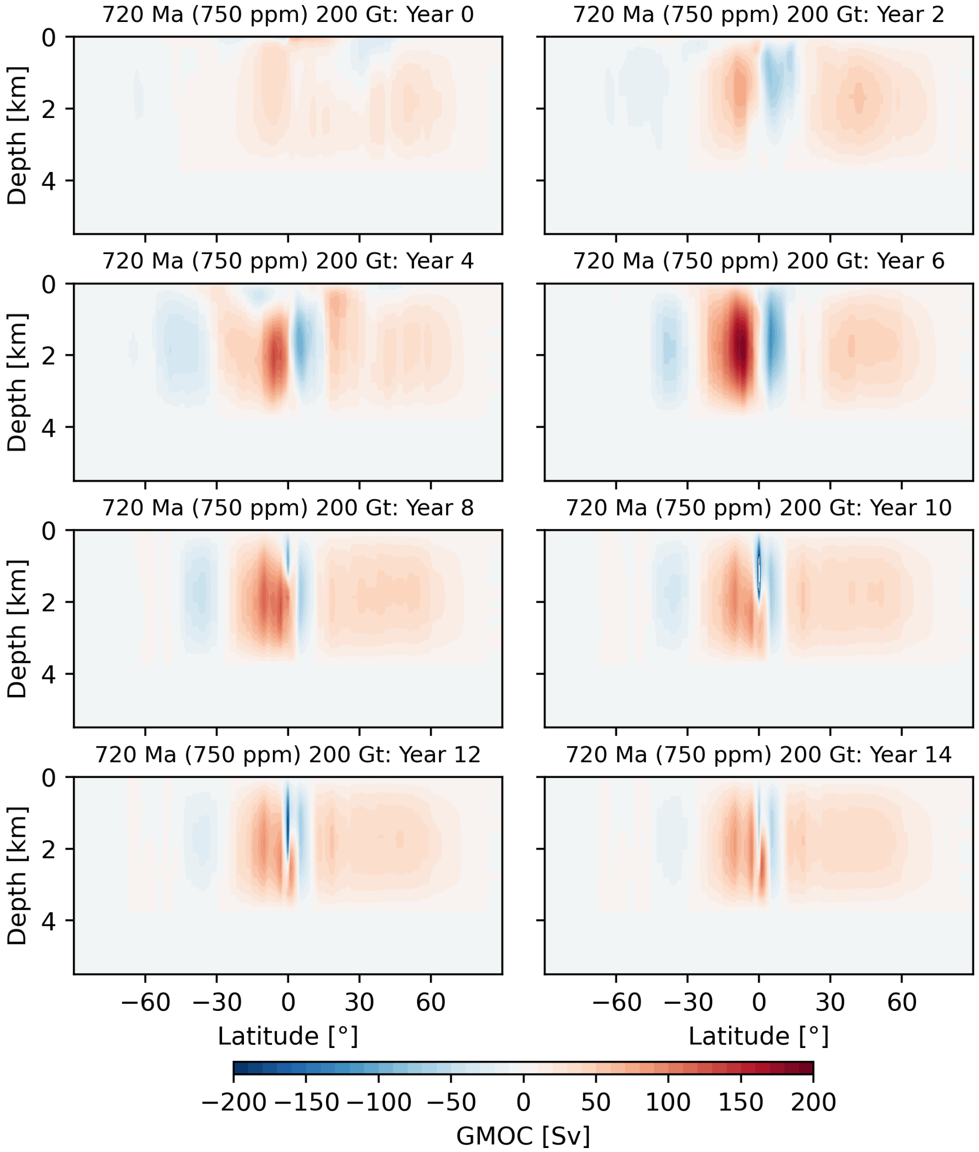


Figure S11: **Eulerian streamfunction for the global meridional overturning circulation, shown as a function of latitude and depth, following the 200 Gt radiative forcing scenario applied to the 720 Ma (750 ppm) climate.**

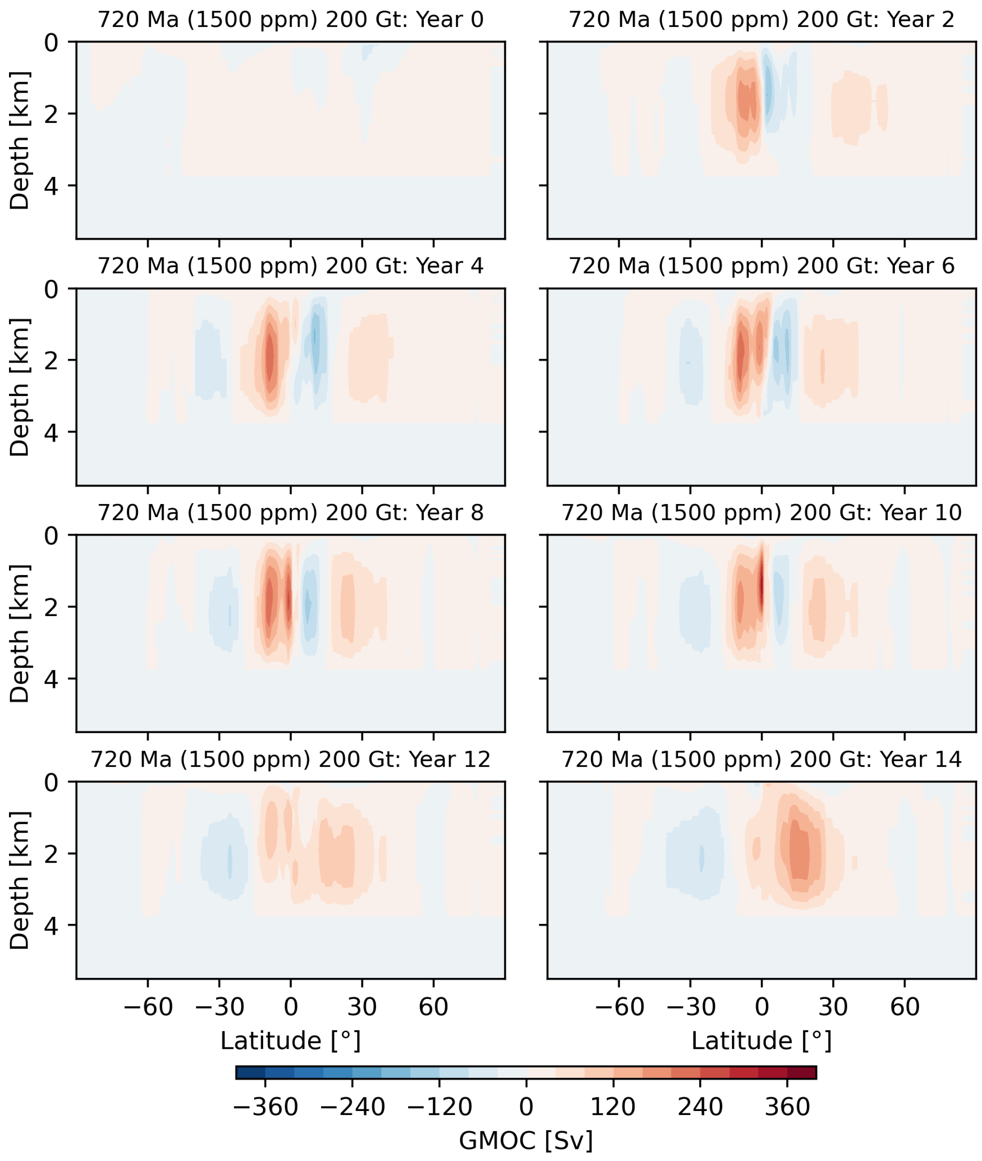


Figure S12: **Eulerian streamfunction for the global meridional overturning circulation, shown as a function of latitude and depth, following the 200 Gt radiative forcing scenario applied to the 720 Ma (1500 ppm) climate.**

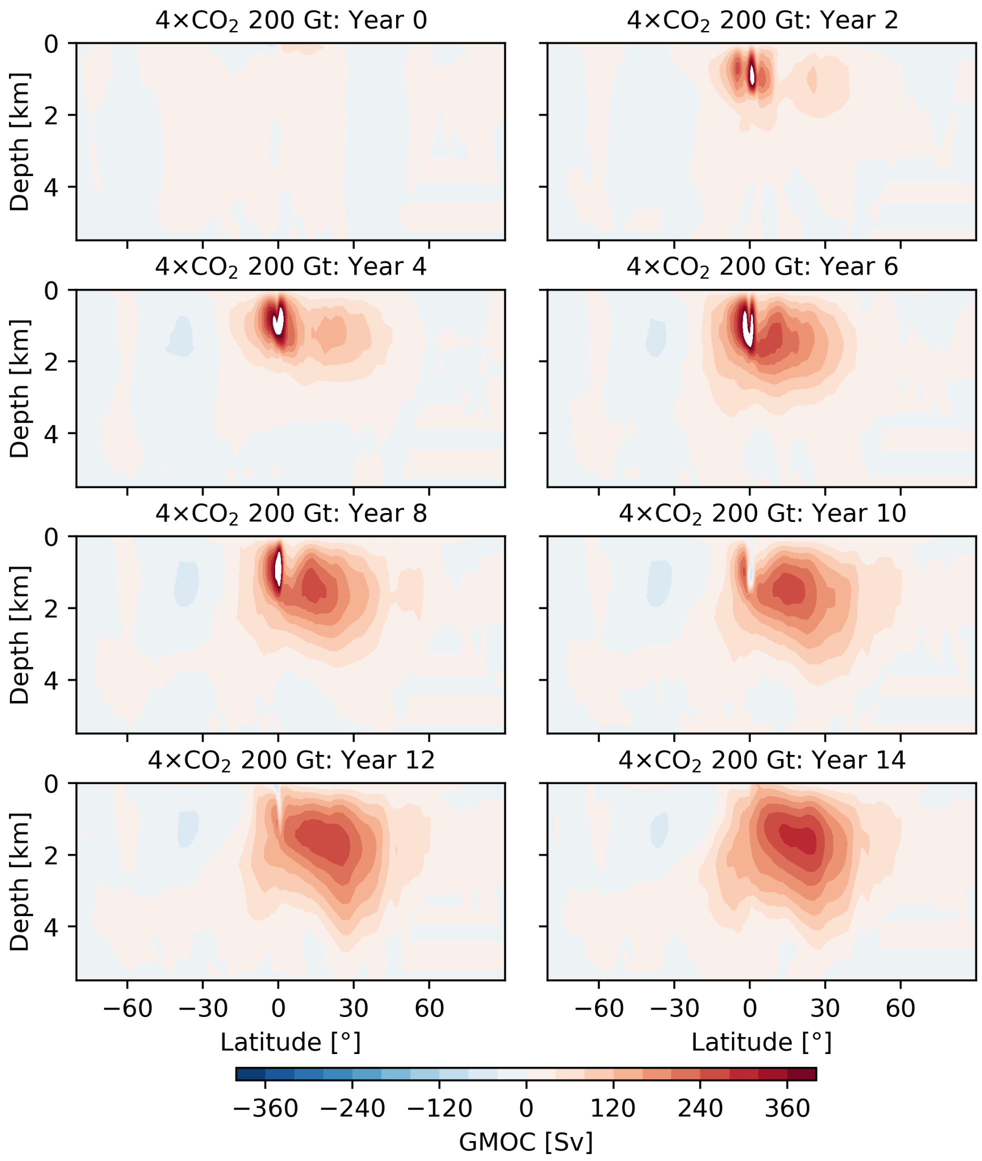


Figure S13: **Eulerian streamfunction for the global meridional overturning circulation, shown as a function of latitude and depth, following the 200 Gt radiative forcing scenario applied to the 4$\times$$CO_2$ climate.**

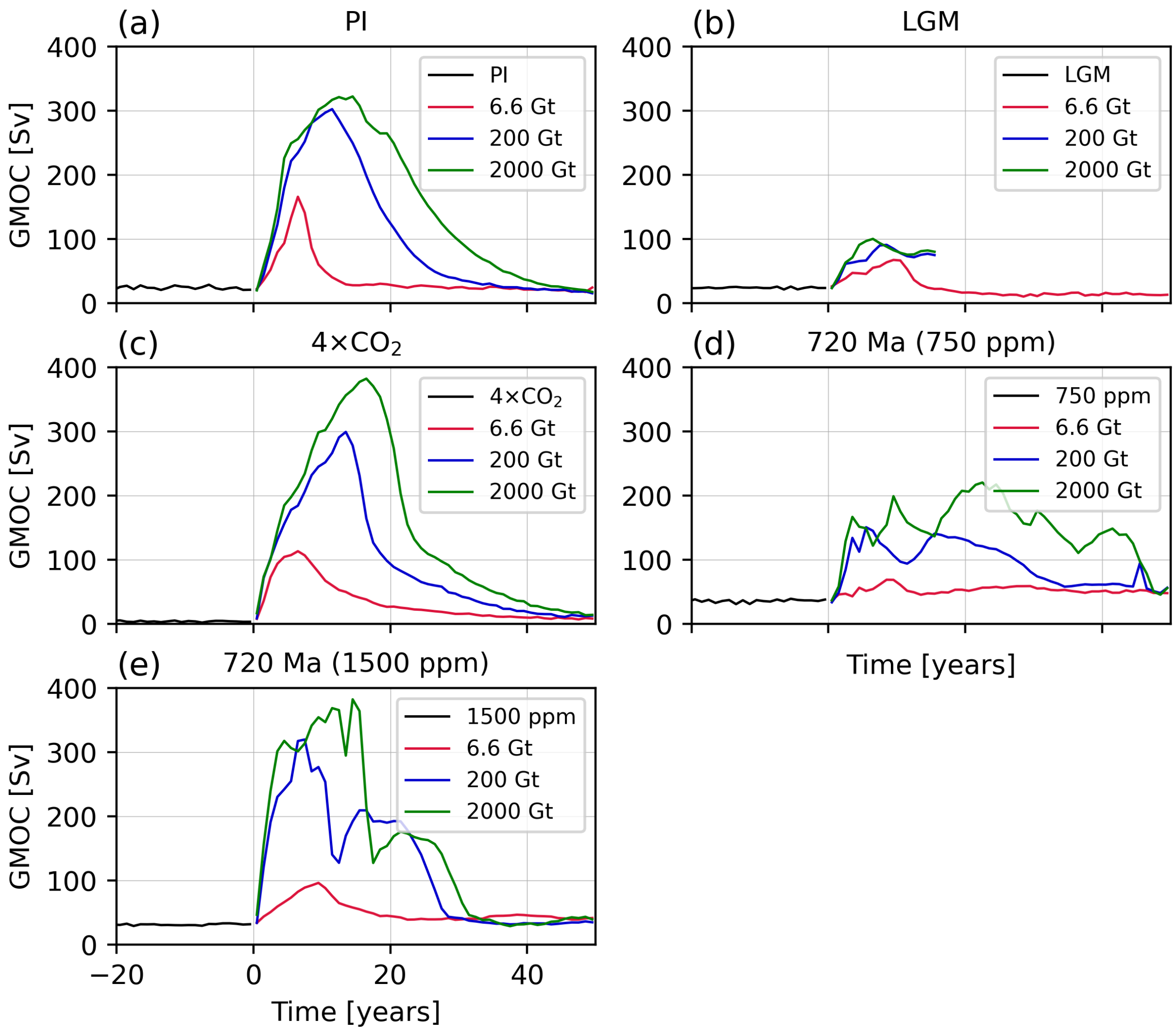


Figure S14: **Response of GMOC strength to the 6.6, 200, and 2000 Gt radiative forcing scenarios.** (a) GMOC strength in the preindustrial simulations. (b) As in (a), but for the LGM simulations. (c) As in (a), but for the 4×$CO_2$ simulations. (d) As in (a), but for the 720 Ma (750 ppm) simulations. (e) As in (a), but for the 720 Ma (1500 ppm) simulations. GMOC strength is computed as the maximum of the annual-mean Eulerian streamfunction below 750m and to the north of 20°N.

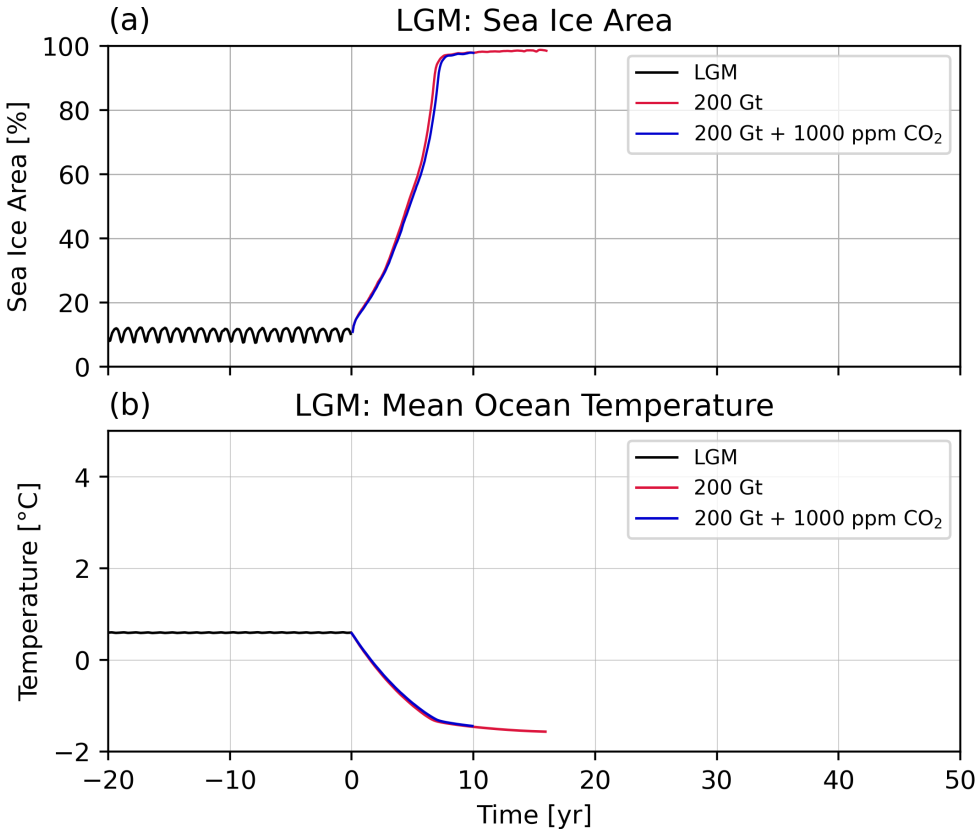


Figure S15: **Response of sea ice coverage and global mean ocean temperature to the 200 Gt sulfate aerosol radiative forcing scenario applied to the LGM climate.** (a) Sea ice coverage in the LGM simulation, with and without an abrupt 1000 ppm $CO_2$ injection. (b) Ocean temperature response in the LGM simulation, with and without an abrupt 1000 ppm $CO_2$ injection.

# Movies

**Movie S1:** Animation of sea ice area for the LGM simulation following the 200 Gt radiative forcing scenario.

**Movie S2:** Animation of sea ice area for the 720 Ma (750 ppm) simulation following the 200 Gt radiative forcing scenario.
